# An Atlas of Plates Illustrative of the Principles and Practice of Obstetric Medicine and Surgery. With Descriptive Letterpress

**Published:** 1840-04

**Authors:** 


Art. X.—1. *An Atlas of Plates Illustrative of the Principles and Practice of Obstetric Medicine and Surgery. With Descriptive Letterpress.* By F. H. Ramsbotham, m.d., Physician to the Royal Maternity Charity, &c. Nos. I. II. and III.—*London*, 1840. 8vo.

2. *Illustrations of Midwifery, a Complete Atlas and Companion to all Obstetric Works.* By M. Ryan, m.d., &c. Parts I.-V.—*London*, 1839-40. 8vo.

These two works being of the same size and price, and nearly on the same plan, look as if they were the speculations of rival publishers, simultaneously suggested by the recognition of an acknowledged want in obstetric literature, and each put forth in the hope of winning the favour of the profession for itself. If this is really the case, the rivalry, we expect, will be such as between Hyperion and a Satyr, or between the Great Western and a Thames lighter; the whole "getting-up" of Dr. Ramsbotham's publication being as elegant as that of Dr. Ryan's is the reverse. "The Atlas" is, indeed, not only one of the neatest but one of the cheapest books we have met with for a long time; each number containing no less than six neatly-engraved steel-plates, besides numerous beautiful woodcuts, and from two to three sheets of handsome letterpress—all for eighteenpence! The "Illustrations," on the other hand, contain only, in each number, four plates, and these wretched specimens of lithography. The subjects of the plates seem, also, as different in conception as they are in execution in the two works. Indeed, several of the figures in Dr. Ryan's work, as, for instance, those in plates

15, 16, 17, 18, seem to us as utterly useless as they are disgusting, and will recall to the inspector the disagreeable impressions excited by some other works of unhappy notoriety published by the same author.

We strongly recommend the work of Dr. Ramsbotham to all our obstetrical readers, especially to those who are entering upon practice. If it proceeds as it has begun, it will be, when completed (announced to be in twelve monthly numbers), not only one of the cheapest, but one of the most beautiful and most useful works in midwifery.

